# A contemporary review of therapeutic and regenerative management of intracerebral hemorrhage

**DOI:** 10.1002/acn3.51443

**Published:** 2021-10-14

**Authors:** Humaira Sadaf, Virendra R. Desai, Vivek Misra, Eugene Golanov, Muralidhar L. Hegde, Sonia Villapol, Christof Karmonik, Angelique Regnier‐Golanov, Dimitri Sayenko, Philip J. Horner, Robert Krencik, Yi Lan Weng, Farhaan S. Vahidy, Gavin W. Britz

**Affiliations:** ^1^ Punjab Medical College University of Health Science Faisalabad Pakistan; ^2^ Department of Neurosurgery Houston Methodist Neurological Institute Houston Texas USA; ^3^ Department of Neurology Houston Methodist Neurological Institute Houston Texas USA; ^4^ Center for Neuroregeneration Houston Methodist Research Institute Houston Texas USA; ^5^ Translational Imaging Center Houston Methodist Research Institute Houston Texas USA; ^6^ Center for Outcomes Research Houston Methodist Research Institute Houston Texas USA

## Abstract

Intracerebral hemorrhage (ICH) remains a common and debilitating form of stroke. This neurological emergency must be diagnosed and treated rapidly yet effectively. In this article, we review the medical, surgical, repair, and regenerative treatment options for managing ICH. Topics of focus include the management of blood pressure, intracranial pressure, coagulopathy, and intraventricular hemorrhage, as well as the role of surgery, regeneration, rehabilitation, and secondary prevention. Results of various phase II and III trials are incorporated. In summary, ICH patients should undergo rapid evaluation with neuroimaging, and early interventions should include systolic blood pressure control in the range of 140 mmHg, correction of coagulopathy if indicated, and assessment for surgical intervention. ICH patients should be managed in dedicated neurosurgical intensive care or stroke units where continuous monitoring of neurological status and evaluation for neurological deterioration is rapidly possible. Extravasation of hematoma may be helpful in patients with intraventricular extension of ICH. The goal of care is to reduce mortality and enable multimodal rehabilitative therapy.

## Introduction

Intracerebral hemorrhage (ICH) affects over 1 million people annually and is associated with extremely high mortality and morbidity.^1^, ^2^ The 30‐day ICH mortality is estimated to be around 40%, and a large proportion of this mortality burden is experienced early during disease. Furthermore, up to 80% of ICH survivors fail to achieve pre‐morbid levels of functional independence.^3^ Therefore, ICH has one of the highest disability‐adjusted life years (DALYs) burdens among all neurological conditions.^4^ A large meta‐analysis indicates little to no change in either ICH incidence or mortality across prior decades.^5^ Additionally, the global burden of disease studies report a 47% absolute increase in the number of ICH cases between 2000 and 2010, coupled with a disproportionally high ICH mortality in low‐to‐middle income countries.^4^ For the United States, some reports indicate a relative decline in ICH mortality^6^; however, others demonstrate a steady mortality trend.^7^ These disparities probably reflect regional, racial, and sex heterogeneity in ICH incidence and mortality.

The etiology of spontaneous (non‐traumatic) ICH is primarily attributed to either hypertensive or amyloid angiopathy. However, recent data additionally suggest that greater adoption of anticoagulation therapies for atrial fibrillation may also contribute to the increasing incidence of coagulopathic ICH. Hypertensive ICH is typically associated with infra‐tentorial or deep supratentorial locations, with higher mortality than amyloid ICH. A “possible” or “probable” amyloid ICH more prevalent among the elderly has a superficial (cortical/hemispheric) location associated with recurrent hemorrhages. There is, however, emerging pre‐clinical evidence that advanced hypertensive angiopathy may play a role in the development of cerebral amyloid angiopathy, and that the two small vessel entities represent a common pathological spectrum.^8^

For many years, there was virtually no effective treatment for ICH, aside from supportive care.^9^ Over time, clinical trials have examined a wide range of medical and surgical prospects for improving outcomes among ICH patients.^10^ Though there are no Class I Level A treatment modalities for ICH patients, advances in early diagnosis, efficient triage, and neurocritical care have likely helped improve survival.^10^ The current evidence suggests that the overall improvement in ICH outcomes is secondary to a multimodal approach of providing care in specialized stroke units, mitigating complications such as venous thromboembolism, controlling hypertension, regulating blood glucose, and appropriately selecting patients for hemostatic therapy, surgical interventions, and neuroprotective agents.^11^

## Medical Management

ICH, as a “neurological emergency,” warrants “rapid diagnosis and management.”^12^ ^(p237)^ The primary objective of pre‐hospital management is to provide basic life support, in the form of airway management and cardiovascular support, and rapid transport to the closest acute stroke facility.^13^ Currently, ICH patients are optimally managed by an interdisciplinary team that includes a neurologist, neurosurgeon, and a neuro‐intensivist specialized in stroke units available 24 h a day.^12^ Studies have shown that admission to a dedicated stroke unit increases the survival likelihood and promotes functional recovery.^14^ Neuroimaging modalities, such as non‐contrast CT scan, can readily differentiate between ischemia or hemorrhage as the cause of neurologic decline.^12^ Certain patient characteristics such as younger age, female sex, nonsmokers, lobar ICH, intraventricular extension, and absence of hypertension or coagulopathy should trigger a workup for the evaluation of underlying vascular abnormalities.^15^, ^16^ Also, approximately one‐third of acutely presenting ICH patients continue to bleed and undergo hematoma expansion, which is associated with poor outcomes. Therefore, early hematoma expansion prediction can help in triage and targeted management. Several radiological markers based on non‐contrast CT scans (baseline hemorrhage volume, irregular hemorrhage shape, satellite sign, island sign, swirl sign, blend sign, black hole sign, density heterogeneity, sedimentation levels, and hypodensities) or on CT angiography (spot sign, spot and tail sign, leakage sign, blush sign, iodine concentration) or on CT perfusion imaging such as dynamic spot sign have been evaluated for their validity toward identifying hematoma expansion among ICH patients.^17^ When available, these markers are recommended to be included in the early evaluation of ICH patients.

## Early Neurocritical Care of ICH Patients

A large proportion of ICH patients are neurologically and hemodynamically unstable, particularly during the early phase after onset. The American Heart Association/American Stroke Association (AHA/ASA) ICH management guidelines recommend that “initial monitoring and management of ICH patients should take place in an intensive care unit [ICU] or dedicated stroke unit with physician and nursing neuroscience acute care expertise (Class I; Level of Evidence B).”^18^ ^(p2041)^ This evidence had been revised in the current iteration (2015) of AHA/ASA guidelines primarily based on data analyzed for in‐hospital mortality among ICH patients managed across a sample of ICUs, compared to those managed at two neuro ICUs.^19^ Similar evidence of reduced in‐hospital mortality also exists for ICH patients managed at certified Comprehensive Stroke Centers (CSCs).^20^ However, specific aspects of neuro ICU care or higher level of care that may directly benefit ICH patients have not been formally evaluated. A single‐center study did not demonstrate a direct impact of a neuro intensivist appointment on outcomes of ICH patients.^21^ It is likely that a combination of consistently achieved quality of care metrics (including aspects of nursing care) results in improved ICH outcomes.^22^, ^23^ This evidence had Gaps between practice and evidence‐based guidelines in the management of ICH patients have been highlighted in the literature.^24^

Similarly, criteria for initial care of ICH patients in neuro ICUs versus dedicated stroke units have also not been fully evaluated. A national survey of emergency medicine physicians in 2007 indicated that only 30% of hospitals had a clearly defined critical pathway for the management of ICH patients (compared to 78% of hospitals with pathways for ischemic stroke patients).^25^ This evidence had With an increasing number of hospitals gaining stroke certification, a greater proportion of hospitals may now have well‐defined management pathways for ICH patients. However, there is likely to be considerable regional variation in decisions to provide acute care to ICH patients. Current accreditation standards for stroke certification in the United States are heavily centered on the acute management of ischemic stroke patients and have a dire need to formally incorporate metrics of triage and care for patients with ICH. Intracerebral hemorrhage patients with extra ventricular drain placement, those requiring active monitoring of intracranial pressure, or with the need for invasive ventilatory support, low Glasgow Coma Score (GCS), risk of hematoma expansion, cerebral compression, or those at risk of malignant cerebral edema may require critical care in a neuro ICU. Moreover, patients with a non‐compressing supratentorial hemorrhage, with smaller ICH volumes, without intraventricular hemorrhage or respiratory failure, with favorable GSC score and systolic blood pressure <200 mmHg may be managed in a dedicated stroke unit.^26^

The mainstay of ICH neurocritical management includes the stabilization of airway, breathing, and circulation (ABCs) to prevent secondary injury from hypoxemia, hypertension, and hematoma expansion. Patients with significant respiratory distress and / or GCS ≤8 require intubation for airway protection and supplemental O_2_ is required to keep O_2_ This evidence had saturation >92%.^10^ It is estimated at 2% of ICH patients experience myocardial infarction, whereas overall 4% encounter a serious cardiac complication which may include ventricular fibrillation, acute cardiac failure or a sudden cardiac death within 48 h of ICH onset.^27^, ^28^ Therefore, continuous cardiac monitoring of ICH patients during the acute phase is routinely recommended.

### Blood pressure control

Studies have found associations between increased hemorrhage growth and high arterial pressure values in ICH patients.^29^ Two recent randomized clinical trials (RCTs), INTERACT‐ II (Intensive Blood Pressure Reduction in Acute Cerebral Hemorrhage Trial – II)^30^ and ATACH‐II (Antihypertensive Treatment of Acute Cerebral Hemorrhage II trial),^31^ provide data informing blood pressure management during the acute phase of ICH.

The INTERACT‐II trial recruited spontaneous ICH patients with high systolic blood pressure (SBP), defined as 150 to 220 mmHg, to receive antihypertensive medications within a 6‐h window after symptom onset. However, disability and mortality rates were not significantly lower in the intensive SBP‐reduction group with a target SBP of <140 mmHg within 1 h, when compared to a target SBP of less than 180 mmHg. ATACH‐II attempted to intervene on SBP in an earlier time window (4.5 h) after symptom onset. Though the incidence of hematoma expansion was lower in the intensive treatment group, this was not statistically significant, nor were differences in morbidity and mortality at 3 months.^30^ Overall, the current evidence is supportive of the safety and feasibility of early intensive SBP lowering.^32^ However, the efficacy of rapid and intensive SBP lowering is yet to be demonstrated. A personalized approach in the management of SBP may be warranted. For example, identifying patients with higher risk of hematoma expansion could determine the need for early intensive BP lowering. Conversely, the presence of diffusion‐weighted imaging (DWI) lesions has been found to be more prevalent among ICH patients who underwent acute SBP lowering and are associated with poor outcomes.^33^, ^34^ Therefore, intensive and rapid SBP lowering among DWI‐positive patients including those with impaired renal function may not be suitable.

Nevertheless, the 2015 AHA/ASA guidelines recommend early BP reduction with SBP targets consistent with those used in INTERACT‐II and ATACHI‐II.^18^ Likewise authors have considered it reasonable to reduce BP more aggressively for higher initial SBP (>220 mmHg) and monitor accordingly.^32^ There are emerging data highlighting the association between early SBP variability and long‐term functional outcomes among ICH patients,^35^, ^36^ but the quantification and control of high SBP variability among critically ill ICH patients needs to be investigated further.

### Hemostatic therapy

Coagulopathy, including medication‐induced coagulopathy, is associated with hematoma expansion and poorer outcomes.^37^ The AHA/ASA guidelines, endorsed by the Neurocritical Care Society and the Society of Critical Care Medicine, therefore included guidance on avoiding or mitigating the adverse effects of antithrombotic agents in patients with ICH.^18^ Various strategies for reversal of coagulopathies associated with specific anti‐coagulants have been summarized by Dastur & Yu,^10^ and more recently by Gulati et al.^38^

For patients who develop ICH, these guidelines provide dosage reversal schedules for unfractionated heparin, low‐molecular‐weight heparin (LMWH) if prescribed in therapeutic doses, and thrombolytics (with cryoprecipitate or an antifibrinolytic agent).^10^ Platelet transfusion is also covered, i.e., as contraindicated for patients taking antiplatelet agents but “considered for patients with aspirin‐ or adenosine diphosphate receptor (ADP) inhibitor‐associated ICH who will undergo a neurosurgical procedure.”.^10^ ^(p25)^

For vitamin K antagonists (VKAs) such as Warfarin‐related ICH, when available, 4‐factor (factors II, IX, X, and VII) or 3‐factor (factors II, IX, X) prothrombin complex concentrates (PCC) are ideally recommended. PCC dosing should be weight‐based and adjusted according to admission INR and type of PCC used. If PCCs are unavailable or contraindicated, rapid alternative treatment with Fresh Frozen Plasma (FFP) at 10–15 mL/kg IV and vitamin K 10 mg IV is recommended.^38^

For ICH associated with Direct Thrombin Inhibitor (DTI) Dabigatran, the use of idarucizumab (5 g IV in two divided doses) is recommended. Hemodialysis may be considered if idarucizumab is unavailable. Furthermore, activated PCC or 4‐factor PCC (50 U/kg) may be considered in dabigatran‐associated ICH when idarucizumab is not available or when ICH is associated with non‐dabigatran DTIs or with direct factor Xa inhibitors (FXa‐Is) (such as rivaroxaban and apixaban). Activated charcoal (50 g) can also be used if the most recent dose of dabigatran, apixaban or rivaroxaban was within 2 h.^38^

A phase 3 RCT tested the efficacy and safety of rFVIIa (20 and 80 µg) for restricting hematoma growth among non‐OAC ICH patients.^39^ Though both doses demonstrated a significant reduction in hematoma growth, there were no differences in the proportion of patients with poor clinical outcomes between the treatment and placebo arms. Furthermore, the incidence of thromboembolic serious adverse events was significantly higher in the 80 µg groups versus placebo. Additionally, tranexamic acid (an antifibrinolytic agent) has also been evaluated for its potential hemostatic effect among non‐OAC ICH patients. The tranexamic acid for hyperacute primary intracerebral hemorrhage (TICH‐2)^40^ was a phase 3 RCT in which participants were randomly assigned to either receive intravenous tranexamic acid or placebo, within 8 h of ICH symptom onset. Patients who received tranexamic acid demonstrated lower likelihood of hematoma expansion (25% vs. 29%, *p* = 0.03). However, there was no difference between the two groups for the primary outcome of day‐90 functional status.

### Management of intracranial pressure (ICP) and perihematomal edema

Addressing the “underlying cause” of ICP is important for treating ICH,^12^ ^(p241)^ which includes particular attention for patients with a low Glasgow coma scale (GCS) score (≤8), transtentorial herniation, significant intraventricular hemorrhage (IVH), and hydrocephalus.^32^ Though the current AHA ICH management guidelines recommend maintaining a cerebral perfusion pressure (CPP) of 50–70 mmHg depending on cerebral autoregulation status,^18^ it is important to note that due to the limitations of data from ICH patients, these recommendations are largely derived from traumatic brain injury literature and may not be regarded as standard management parameters for ICH patients.

Placing the head in a neutral position and raising the head‐end of the bed to an angle of 20–30 increases venous return.^12^ Hyperventilation can be used to reach a CO_2_ partial pressure of 28–35 mmHg and maintain it between 25 and 30 if necessary to reduce ICP.^12^ However, the effect of hyperventilation is transient and cannot be relied upon to control ICP for extended periods of time.^12^ Osmotherapy with Mannitol and hypertonic saline (HTS) are also useful in treating ICP.^10^, ^41^ Other proposed strategies to lower ICP include sedation via propofol with short‐acting analgesics (opioids) or other IV drug regimens and induced hypothermia.^42^

### Seizure prophylaxis and treatment

Despite a substantial frequency of electrographic and clinical seizures reported in ICH patients,^32^ there is no evidence demonstrating an association between seizures and poor neurological outcomes or mortality.^43^, ^44^ Therefore, routine prophylactic use of antiepileptic drugs is not recommended in current guidelines. However, continuous EEG monitoring may be reasonable among ICH patients with depressed mental status out of proportion to the degree of brain injury, and patients with demonstrated electrographic seizures on EEG may be treated by anti‐epileptic drugs. There is also limited evidence to support the prophylactic use of anti‐epileptic drugs in patients undergoing craniotomy.^45^

### Glucose management

Hyperglycemia in ICH patients is associated with worse morbidity and mortality.^46^ However, overcompensating with stringent glucose control (i.e., target 80–110 mg/dL) increases the risk of hypoglycemia and likewise increases morbidity and mortality.^47^ Therefore, the current American Heart Association guidelines for ICH management recommend close monitoring of glucose and avoiding both hyper and hypoglycemia.^18^

### Deep vein thrombosis prophylaxis and treatment

ICH patients are at risk for venous thromboembolism (VTE), with 1%–5% rate of symptomatic deep vein thrombosis (DVT).^48^, ^49^ The need to prevent VTE among ICH patients must be weighed against the essential goal of achieving hemostasis and preventing further bleeding. The 2015 Neurocritical Care Society guidelines, citing evidence rated as high quality, strongly recommend initiating intermittent pneumatic compression devices or graduated compression stockings upon admission, with further suggestions (on weaker‐grade evidence) to continue mechanical prophylaxis alongside pharmacological agents, namely prophylactic doses of subcutaneous unfractionated heparin or LMWH in patients with stable hematomas, within 48 h of admission.^50^ In the case of anticoagulation contraindication, inferior vena cava filter placement should be considered.^10^

### Stress ulcer prophylaxis and dysphagia screening

Proton pump inhibitors and histamine‐2 receptor blockers have prophylactic benefits impacting all‐cause mortality in neurocritical care patients, including those with ICH, who are at high risk for upper gastrointestinal bleeding.^51^ To manage complications from pneumonia, a dysphagia assessment should be performed in all patients before oral administration.^18^

## Surgical Management

The cornerstone of surgical management for ICH patients is the reduction of cerebral hematoma volume. This approach hinges on strong evidence that mass removal reduces neurological tissue damage by preventing cerebral herniation and possibly by alleviating local ischemia. Furthermore, hematoma removal is also associated with a reduction in blood product‐induced excitotoxicity and neurotoxicity.^52^

The Surgical Trial in Lobar Intracerebral Hemorrhage I and II (STICH I and II) trials did not show clinical benefit for early surgical removal of ICH when compared with best medical management plus delayed surgery if indicated.^53^, ^54^

Due to the equivocal results of the STICH trials, especially regarding deeper hemorrhages, minimally invasive techniques for hematoma evacuation emerged in the current decade^55^; minimally invasive procedures can be performed under local anesthesia and reduce operative time and surgical trauma compared to craniotomy.^42^ Meta‐analyses have corroborated the superiority of minimally invasive techniques and endoscopic approaches.^56^, ^57^ A study of 465 patients^58^ (cited in AHA/ASA guidelines^18^), comparing needle aspiration of basal ganglia hemorrhages (25–40 mm^3^) to medical management alone, found no significant impact on mortality but did find the improved neurological outcome in the aspiration group at 3‐month follow‐up.^18^, ^58^ Hence, the guidelines conclude that minimally invasive techniques for clot evacuation may be promising.^18^

A multicenter RCT MISTIE II (Minimally Invasive Surgery Plus rt‐PA for Intracerebral Hemorrhage Evacuation)^59^ was conducted to test the safety and efficacy of hematoma evacuation after ICH. The image‐guided catheter‐based removal of the blood clot was done in subjects with hypertensive ICH. There was a positive correlation between perihematomal edema reduction and percent of ICH removed. Still, no difference in the primary outcome was found between minimally invasive surgery plus alteplase group and the standard medical care group. Subsequently, MISTIE III was designed to confirm MISTIE II preliminary findings in a larger number of patients.^60^ It was aimed to assess the effect of decreasing clot size to 15 mL or less, on functional outcome among ICH patients. MISTIE led to a 69% mean reduction in hematoma size compared with the standard medical therapy group, but more than 40% of patients did not reach the goal of ≤15 mL. For secondary outcomes, there was no benefit in terms of the extended Glasgow Outcome Scale at 1 year (adjusted risk difference 4·2%, *p* = 0.28), but mortality was lower in the MISTIE group at 7 days and 1 year (HR = 0.67, *p* = 0.037). In addition, an as‐treated analysis focused on the MISTIE patients who achieved a hematoma size of 15 mL or less showed a benefit of the procedure by 10.5% (*p* = 0.03) for functional outcomes (mRS 0–3) at 1 year.

A Stereotactic CT‐guided Endoscopic Surgery arm was added to the MISTIE trial (MISTIE ICES trial), with positive outcomes and few complications.^61^ Based on a similar approach, a multicenter RCT comparing standard medical management to early (<24 h) surgical intervention using minimally invasive parafascicular surgery (MIPS) [BrainPath^®^] is currently underway.^62^ Results from these ongoing trials are likely to provide important evidence on optimal surgical endpoints, patient selection, and timing of surgery. Despite promising primary and secondary outcomes of these studies, further RCTs are required to evaluate the clinical benefit and application of various minimally invasive procedures in targeted ICH patient populations.

### Management of intraventricular hemorrhage and hydrocephalus

The CLEAR III trial (***Clot Lysis***: Evaluating Accelerated Resolution of Intraventricular Hemorrhage Phase III)^63^ was conducted to test the benefits of the combination of extra ventricular drainage and low dose alteplase to remove IVH. Subjects received up to 12 doses of recombinant tissue plasminogen activator (rtPA) or 0.9% saline via the extra ventricular drain. There was a 50% decrease in the mortality odds for rtPA versus saline (adjusted OR 0.50, *p* = 0.004), and the frequency of adverse effects was similar in both groups. As the trial was neutral on the primary outcome of functional improvement, the trial authors suggested further research to ensure the efficacy of this rtPA for IVH clearance.

### Repair and regeneration therapy

#### Neuroprotection

Neuroprotection broadly implies “salvage, recovery or regeneration of the nervous system, its cells, structure and function.”^64^ ^(p4)^ The concept of neuroprotective drugs in the management of ICH has recently evolved. Magnesium has a neuroprotective effect via calcium antagonism and potent vasodilation.^65^ However, despite multiple plausible and promising underlying mechanisms which suggest therapeutic effect for ICH patients,^66^, ^67^ RCT on intravenous Mg^++^ have shown no such benefit.^68^

Studies on cerebral glucose utilization have demonstrated that N‐methyl D‐aspartate (NMDA) and α‐Amino‐3‐hydroxy‐5‐methyl‐4‐isoxazolepropionic acid (AMPA) glutamate receptor antagonists can block increased cerebral glucose utilization.^69^ Additional genomic brain studies identified that the Src family member Lyn increased expression over 20‐fold in ICH patients. This line of research has prompted research on the role of Src antagonist PP2 in preventing damage in ICH.^70^

Although the role of iron damage in the perihematomal area and protection by iron chelators is well theoretically established,^71^ trials for the clinical use of iron chelators are yet to demonstrate efficacy. The iDEF trial (Intracerebral Hemorrhage Deferoxamine Trial) was a phase II, double‐blind RCT that evaluated the role of deferoxamine mesylate in clinical outcome among ICH patients at 3 months (defined as modified Rankin Scale (mRS) score of 0–2) compared to placebo.^72^ At day 90, 34% of the study participants in the deferoxamine group and 33% in the control group had mRS scores of 0–2. The investigators concluded that although the use of deferoxamine mesylate is safe, further investigation of deferoxamine mesylate in potentially improving functional outcomes among ICH patients is futile.

#### Stem cell transplant

Stem cells are defined as “undifferentiated cells that retain the capacity to proliferate and produce generations of progenitor cells.”^73^ ^(p101)^ Several factors need consideration for the investigation of stem cells as a potential therapeutic among ICH patients, such as stem cells type, their differentiation status, proliferation capacity, the route and timing of administration, the intended location, irreversibility of treatment, regeneration capacity, and long‐term survival of engrafted cells.^74^ Several small clinical trials have been conducted using bone marrow mononuclear cells (BMNCs) in ischemic and hemorrhagic stroke patients.^73^, ^75^, ^76^, ^77^, ^78^, ^79^ These trials used intravenous (IV), intrathecal (IT), and intracerebral (IC) routes to inject stem cells.

The studies have reported functional improvement at 6–12 months on the NIHHS scale & Rankin scale, a decline in motor deficits assessed by Ashworth's Scale for Spasticity, and improvement in ambulation and equilibrium assessed by the Tinetti scale.

#### Sensorimotor rehabilitation

As in ischemic stroke recovery, augmenting neural reorganization is considered to be one of the primary mechanisms to improve outcomes in patients with ICH.^80^ Similar to ischemic stroke, there is a critical gap in guidance for ongoing rehabilitation practices among ICH patients.^80^, ^81^ More specifically, little is known about the optimal time to initiate therapy, daily dose, frequency, and total duration of treatment post‐ICH to optimize sensorimotor recovery. Moreover, the current application of neuromodulatory approaches in ICH rehabilitation is still in its infancy. Noninvasive brain stimulation (e.g., transcranial direct current stimulation) is under study as a potential adjuvant to therapy in ischemic stroke (Clinical Trial #NCT00909714),^82^ as the underlying mechanisms of this approach look promising.^80^, ^83^, ^84^ Small blinded studies previously conducted have shown a short‐term recovery but with little proof of long‐term benefits.^85^ More extensive studies with heterogeneous ICH phenotypes are, therefore, warranted to establish the efficacy of noninvasive brain stimulation in human patients with ICH.^80^

Currently, rehabilitation services range from inpatient rehabilitation (IRF) to facility‐based or in‐home outpatient rehabilitation, indicated based on the severity of condition and impairments; it is essential to tailor the treatment plan to the motor and cognitive status of each ICH patient.^80^ A comprehensive approach is recommended, considering the potential need for the rehabilitation team to include physical therapists, occupational therapists, and speech and language pathologists, coordinated by a physician to manage all ICH‐related and comorbid complicating factors.^80^

## Secondary Prevention of ICH

The cumulative risk of ICH recurrence is reported to be between 1% and 5% per year.^86^, ^87^ Primary drivers of high recurrence risk are age, hypertension, cerebral amyloid angiopathy (CAA), use of antithrombotics, burden of underlying cerebral small vessel disease (particularly cerebral microbleeds), and genetic polymorphism.^88^, ^89^, ^90^, ^91^, ^92^ Measures to control BP post‐ICH need to be initiated immediately after ICH (summarized in the section above on BP management) and maintenance of long‐term systolic BP of <130 mmHg and diastolic BP of 80 mmHg is considered reasonable.

Among ICH patients with nonvalvular atrial fibrillation on Warfarin, post‐ICH avoidance of long‐term anticoagulation with Warfarin seems to be reasonable.^93^, ^94^, ^95^ There is mounting observational evidence about the superior comparative effectiveness of non‐vitamin K antagonists (NOACs) versus Warfarin in preventing thromboembolic events (including ischemic stroke) and reduced rates of ICH recurrence among ICH patients with atrial fibrillation.^96^ However, data from several ongoing RCTs are awaited to provide conclusive evidence on choice of modality (including the evaluation of left atrial appendage closure) and timing for the resumption of oral anticoagulation among ICH patients.^97^ The absolute risk of recurrent ICH in the general population with the use of antiplatelets appears to be small relative to the number of cardiac and cerebral ischemic events prevented.^98^, ^99^ Therefore, current ICH management guidelines recommend considering antiplatelet monotherapy after any ICH particularly when there is a strong indication for these agents.^18^

Finally, there is insufficient data to recommend restriction on the use of statins in ICH patients. The ongoing international phase 3 RCT “Statin Use in Intracerebral Hemorrhage Patients (SATURN)” is designed to determine whether continuation versus discontinuation of statin drugs after spontaneous ICH would lead to higher ICH recurrence over 24 months.^100^

## Conclusion

Intracerebral hemorrhage has historically been one of the most devastating medical conditions and there continues to be a significant need in establishing it as a treatable condition. However, despite the grim prognosis associated with ICH, there are reasons for optimism. The comprehensive efforts in defining and adhering to the all‐encompassing quality of care metrics in medical management, and significant advancements in neurocritical care, have been the cornerstones of progress. Additionally, neurosurgical advances in minimally invasive techniques, endoscopy, and stereotactic systems have demonstrated promising results of surgery during the last decade. The novel therapeutic potential of neuroprotective agents, neuromodulation approaches, cellular therapy, and neurorehabilitation is also expected to enhance patient outcomes. Given the continued mortality and morbidity burden of ICH, research and development on prevention, acute management, and recovery have to be prioritized at the national and global levels. Large longitudinal cohorts of heterogeneous ICH phenotypes need to be established that enhance our understanding of ICH outcomes in the light of genetic, biological, and radiological markers of recovery. Such infrastructure will also support rapid testing of potential therapeutic modalities and synergetic effects of multimodal approaches. Large pragmatic effectiveness trials, as well as quick adaptive efficacy trials, are concurrently needed to achieve the desired goal of improving long‐term functional, quality of life, and cognitive outcomes among ICH patients.

## Author Contributions

Humaira Sadaf – manuscript drafting and preparation, study design. Virendra R. Desai – critical review of the manuscript and editing, study concept, and design. Vivek Misra – critical review of the manuscript, study concept, and design. Eugene Golanov – critical review of the manuscript, study concept, and design. Muralidhar L. Hegde – critical review of the manuscript, study concept, and design. Sonia Villapol – critical review of the manuscript and editing, study concept, and design. Christof Karmonik – critical review of the manuscript, study concept, and design. Angelique Regnier‐Golanov – critical review of the manuscript, study concept, and design. Dimitri Sayenko – critical review of the manuscript, study concept, and design. Philip J. Horner – critical review of the manuscript, study concept, and design. Robert Krencik – critical review of the manuscript, study concept, and design. Yi Lan Weng – critical review of the manuscript, study concept, and design. Farhaan S. Vahidy – manuscript preparation, critical review of manuscript, study concept, and design. Gavin W. Britz – critical review of the manuscript, study concept, and design.

## Conflicts of Interest

None declared.
